# Evaluation of empirical and mechanistic models and sampling intervals to model the lactation curves of F1 dairy sheep

**DOI:** 10.1007/s11250-025-04365-z

**Published:** 2025-03-14

**Authors:** Lilian Guevara, Nicolas Lopez-Villalobos, Geoffrey Ernest Pollott, Benito Albarrán-Portillo, Alberto Magno Fernandes, Jesús Armando Salinas-Martínez, Juan Carlos Angeles-Hernandez

**Affiliations:** 1 Centro de Ciências E Tecnologias Agropecuárias, Universidade Estadual Do Norte Fluminense, Campos Dos Goytacazes, 28013-620 Brazil; 2 School of Agriculture and Environment, Massey University, Private Bag 11 222, Palmerston North, 4442 New Zealand; 3 Royal Veterinary College, Royal College Street, London, NW1 0TU UK; 4 Centro Universitario UAEM - Temascaltepec, Universidad Autónoma del Estado de México, 51300 Temascaltepec, Mexico; 5 Instituto de Ciencias Agropecuarias, Universidad Autónoma del Estado de Hidalgo, México, Santiago Tulantepec de Lugo Guerrero, Hidalgo, 43600 México; 6 Departamento de Medicina y Zootecnia de Rumiantes, Facultad de Medicina Veterinaria y Zootecnia, Universidad Nacional Autónoma de México, Ciudad de México, 04510 México

**Keywords:** Dairy ewe, Mathematical modeling, Lactation curve, Milk recording

## Abstract

Recently Latin American countries have developed a dairy sheep industry with an increasing number of specialized dairy-sheep flocks. The objective of this study was to evaluate the goodness of fit of empirical and mechanistic models and sampling interval to model the lactation curve of F1 dairy sheep with different shape of lactation curves in an intensively managed flock of the central highlands of Mexico. A total of 4,494 weekly (7D) test day records (TDR) from 156 lactations were analyzed. Three datasets were generated from the original 7D data set, setting TDR at different sampling intervals: 14 (14D), 21 (21D) and 28 (28D) days. Lactation curves were fitted using two empirical (Wood and Wilmink) and two mechanistic models (Dijkstra and Pollott). The parameters of the empirical and mechanistic models were estimated using the iterative non-linear curve fitting procedure in R. The results showed that the estimation of TMY was not affected by the sampling interval. However, the estimation of peak yield (PY) and day at peak yield (TPY) was affected by sampling interval (*P* < 0.05), with better estimates for 7D and 14D. Estimates of PY and TPY differed between the empirical and mechanistic models with both methods failing to estimate PY and TPY in atypical curves. The Dijkstra model showed the best fit for typical curves and the Pollott model for atypical curves in all the sampling intervals evaluated.

## Introduction

Sheep milk production plays an important economic role in some high-income countries, mainly in the Mediterranean region, due to the high prices of dairy products, mainly cheese (Boyazoglu and Morand-Fehr 2001; Hoffman and Gerber 2013; Angeles-Hernandez et al. 2016). In the last two decades, Latin American countries have increased the production of sheep milk (15.7%) and cheese (2.8%) (FAOSTAT 2024) with the use of specialized dairy sheep in Mexico (Angeles-Hernandez et al. 2018a) and Brazil (Brito et al. 2006; Bianchi 2018). However, there is little information on the productive performance of the breeds used under the environmental conditions of these countries, which limits the implementation of improvement strategies (Angeles-Hernandez et al. 2018a).

The lactation curve is the representation of milk production over time and can be described by different mathematical models (Morant and Gnanasakthy 1989). There are several criteria for classifying mathematical models of the lactation curve. Depending on the level of approximation of the biological processes involved and the organizational hierarchy, these functions can be classified as empirical or mechanistic (France and Kebreab 2008). Empirical models are used to find the mathematical equation that best fits the experimental data and are based on direct observation of the biological process (Giraldo et al. 2002; Pulina and Bencini 2004). On the other hand, a mechanistic model is developed to understand the components of the system with a biological interpretation of its parameters (Pulina and Bencini 2004; Angeles-Hernandez et al. 2021).

The ability of mathematical models to fit the lactation curve of animals in a population is influenced by several factors such as animal species (Dijkstra et al. 1997), production system (Cunha et al. 2010), shape of the lactation curve (Angeles-Hernandez et al. 2014a, b), day to first test-day record (TDR) (Schaeffer and Jamrozik 1996), number of TDR (Anderson et al. 1989; McGill et al. 2014) and sampling intervals (Schaeffer and Jamrozik 1996; Wasike et al. 2011; McGill et al. 2013). There is evidence that mechanistic models are more sensitive to sampling interval than empirical models (Pollott 2000). According to ICAR (2018), the standard sampling interval for recording sheep milk is called "A4", which implies an interval of 30 ± 3 days between TDR. From a logistical and economic point of view, each milk recording represents extra labor and cost for producers; however, an insufficient number of records can have a detrimental effect on the ability of lactation curve models to fit the lactation curves and predict total yield with high accuracy.

Some studies have shown the effect of sampling interval on the goodness of fit of lactation curve models in cows (Silvestre et al. 2006; Wasike et al. 2011; Ghosh and Khan 2014; Sitkowska et al. 2020), goats (Landi et al. 2021) and sheep (Pollott 2000). However, no studies have focused on identifying the most appropriate model and sampling interval for modelling the lactation curves of dairy sheep under the climatic conditions of Latin American countries. Estimating the parameters describing the lactation curve of individual animals is important to characterize the performance of imported specialized dairy sheep breeds in the different environments where they have been developed. This knowledge is necessary for the design of nutritional, management and health strategies that support the expression of the genetic potential of the imported breeds. Therefore, the objective of this study was to evaluate the goodness of fit of empirical and mechanistic models and sampling intervals to model the lactation curve of F1 dairy sheep with different shape of lactation curves in an intensively managed flock of the central highlands of Mexico.

## Materials and methods

### Data

This research was carried out in an organic dairy herd of sheep located in the municipality of Marques, Querétaro, Mexico. The lactation curves of F1 dairy sheep of the following breeds were analyzed: East Friesian (sire line) and Pelibuey, Suffolk and Black Belly (dam line). Ewes were milked on the fifth day after lambing, mechanically milked once a day and milk yield recorded once a week. Only lactations with the following information were included in the analysis: ewe identity, lambing date, number of lambing and number of lambs. The ewes were grazed in strips on mixed swards of ryegrass (*Lolium multiflorum*) and lucerne (*Medicago sativa*), supplemented with lucerne hay and maize grain at milking.

A total of 4,494 weekly (7D) TDRs from 156 lactations from all seasons of 2015 were analysed. On average each lactation had 29.3 ± 13 weekly TDR and range of the lactation length was from 70 to 517 days with the first TDR at day 15.3 ± 12.4. Three datasets were generated from the original 7D data set, keeping TDR at different sampling intervals: 14 (14D), 21 (21D) and 28 (28D) days. The last interval of 28 days corresponds to the A4 method proposed by ICAR as the standard method for milk recording in dairy sheep.

Total lactation milk yield (TMY) was computed using the Fleischmann method, often called the centering date method (Sargent 1968):

1 $$TMY={y}_{1}\times {t}_{1}+{\Sigma }_{i}((({y}_{i}+{y}_{i}+1)/2)\times {D}_{i})+{y}_{k+1}\times 7$$

where TMY is the total milk yield, y_1_ is milk yield at first TDR, t_1_ is the interval in days between lambing and first TDR, y_i_ is the yield at recording *i* (*i* = 1,…k) and D_i_ is the interval between the record *i* and record (*i* + 1), and 7 is the interval in days between the last TDR and drying-off. The lactation curve peak yield (PY) and time to peak yield (TPY) traits were obtained individually from the estimated curve parameters for each ewe.

### Lactation curve models

Lactation curves were individually fitted using two empirical models (Wood and Wilmink) and two mechanistic models (Dijkstra and Pollott):

*Wood model or gamma incomplete model* (Wood 1967):

2 $${y}_{t}=a{t}^{b}{\text{exp}}^{-ct}$$

where y_t_ is the milk yield at time t, *a* is the parameter representing the milk yield at the beginning of lactation and *b* and *c* are the parameters of the increasing and decreasing slopes of the lactation curve before and after peak production, respectively.

*Wilmink model or Wilmink’s exponential model* (Wilmink 1987):

3 $${y}_{t}=a+bex{p}^{-kt}+ct$$

where y_t_ is the milk yield at time t, *a* parameter is associated with the level of milk production, and *b* and *c* parameters represent the increase and decrease in milk production around peak lactation, respectively. The *k* parameter has a fixed value derived from a preliminary analysis and is associated with the time of peak yield; for the current study the value of the *k* parameter was 0.033.

*Dijkstra model* (Dijkstra et al. 1997):

4 $${y}_{t}=a exp ((b/c)\times (1-exp(-ct))-(dt)$$

where y_t_ is the milk yield at time t, *a* is the theoretical initial milk yield, *b* is the specific rate of secretory cell proliferation at parturition, *c* is the decay parameter and *d* is the specific rate of secretory cell death.

*Pollott model* (Pollott 2000):

5 $${y}_{t}=(a/(1+((1-0.9999999) / 0.9999999) * exp (-b \times (t-150)))) \times (2 - exp (c\times t))$$

where y_t_ is the milk yield at time t, *a* is the maximum milk secretion potential of the lactation, *b* is the relative rate of increase in the number of secretory cells during early lactation, *c* is the relative rate of decrease in the number of cells. In the current study, a reduced version of the Pollott (2000) model was used to test models with a similar number of parameters.

The different shapes of lactation curves were defined for each model according to the value of their parameters. For the Wood model, a typical curve was defined when the parameters *b* and *c* had positive and negative values, respectively (*b* > 0 and *c* < 0). For this model, other combinations of *b* and *c* represent an atypical curve. In the Wilmink model, the lactation curve had a typical shape when *b* and *c* were negative (*b* < 0 and *c* < 0). The other parameter combinations of *b* and *c* described an atypical curve (Macciotta et al. 2005). For the Dijkstra and Pollott models, the shape of lactation was identified individually by visual inspection of the plots of predicted values at each day in milk.

The parameters of the empirical and mechanistic models were estimated by the iterative nonlinear curve fitting procedure of regression analysis using the ‘nlsLM’ function of the ‘minpack.lm’ package (Elzhov et al. 2022) in the R environment for statistical computing (version 4.0.2; R Core Team 2022). For each lactation, the convergence criterion was reached when the difference between the sum of squares errors of two consecutive iterations was less than 10^–6^. Lack of convergence of each lactation curve model was considered to have occurred when 100 iterations were completed without a reduction in the sum of squares error. For each sampling interval, the percentage of convergence of the four lactation curve models was calculated. The effect of sampling interval and shape of lactation curve for each mathematical model on TMY, PY, TPY and goodness of fit criteria were evaluated using a GLM procedure in the R environment for statistical computing (version 4.0.2; R Core Team 2022).

### Goodness of fit

Goodness of fit in each lactation was evaluated using:

Mean square of prediction error (MSPE), Root-mean-square prediction error (RMSPE):

6 $$\text{RMSPE}= \sqrt{MSPE}$$

Akaike´s Information Criterion (AIC) calculated as:

7 $$AIC = n \times LL + 2 \times p$$

where *n* is the number of TDR of the lactation under evaluation, LL is the log-likelihood for the model using the natural logarithm (e.g. the log of the MSPE), and *p* is the number of parameters in the model under evaluation.

Bayesian´s Information Criterion (BIC) calculated as:

8 $$BIC = n \times LL + p \times log(n)$$

where log() is the natural logarithm.

Finally, the coefficient of correlation between (r) the actual TMY (estimated from the Fleischmann method) and the estimated TMY was calculated.

## Results

### Lack of convergence

**Fig. 1:**
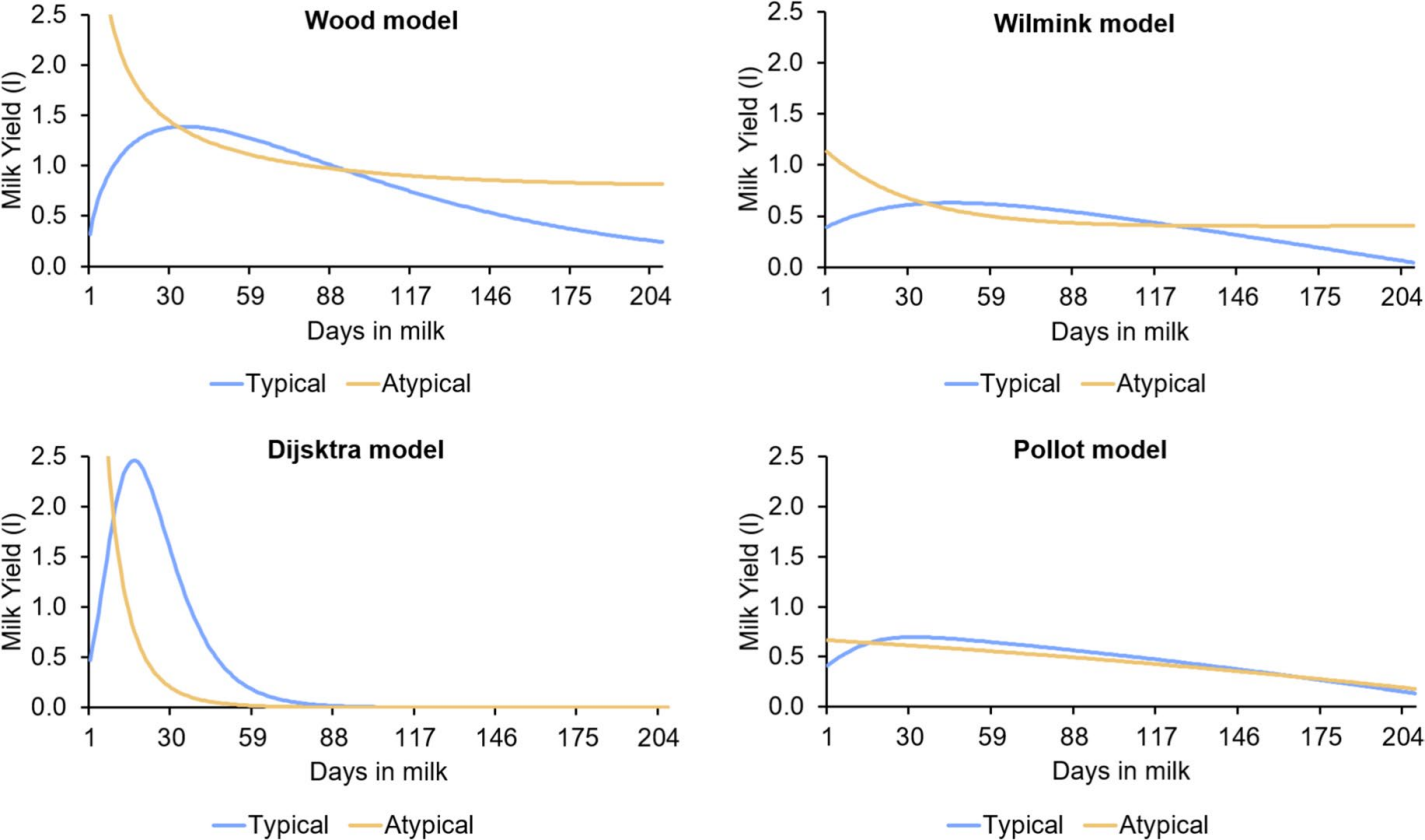
Typical and atypical dairy sheep lactation curves fitted using empirical (Wood and Wilmink) and mechanistic (Dijkstra and Pollott) models

The first finding of the study was related to the two different shapes of lactation curves exhibited by F1 dairy ewes, typical and atypical (Fig. 1). Using the Fleischmann method and all 7D test-day records of all 156 lactations, 88 were classified as atypical curves representing 56.4% of the total. The number of lactation curves that reached convergence according to the various models, shapes and sampling intervals is shown in Table 1. The Wilmink and Dijkstra models were able to detect a higher number of atypical curves across all sampling intervals than the other two. Failure of the iterative procedure or lack of convergence were higher with increasing sampling interval in all models, with a greater effect shown by the Wood and Wilmink models. Regarding the convergence ability of the empirical and mechanistic models, the Pollott model showed a higher frequency of convergence failures in all sampling interval (> 12.8%).

**Table 1:** Number and percentage of converged lactation curves by shape of the lactation curve and milk sampling interval (*n* = 156)

	Typical				Atypical				Converged fitting curves (%)			
	7	14	21	28	7	14	21	28	7	14	21	28
Wood	68	70	69	65	82	80	76	67	150 (96.2)	150 (96.2)	145 (92.9)	132 (84.6)
Wilmink	58	58	62	52	98	97	88	84	156 (100)	155 (99.4)	150 (96.2)	136 (87.2)
Dijkstra	60	63	58	59	96	91	93	93	156 (100)	154 (98.7)	151 (96.8)	152 (97.4)
Pollott	61	53	56	57	75	74	75	72	136 (87.2)	127 (81.4)	131 (84.0)	129 (82.7)

### Shape of lactation curve

Measures of goodness of fit to evaluate the ability of empirical and mechanistic models to mimic typical and atypical lactation curves of dairy sheep under different sampling intervals are presented in Tables 2 and 3. The shape of the lactation curve had a significant effect on the estimation of lactation traits (P < 0.001). The results.

**Table 2:** Goodness of fit measures of two empirical models using different sampling intervals to model the lactation curves of F1 dairy sheep

	Wood															
	Typical							Atypical						***P***-value		
Item^1^	Actual		7D	14D	21D	28D	SEM	Actual	7D	14D	21D	28D	SEM	Interval	Shape	Interval × Shape
TMY	106.7		104.5	101.3	104.5	115.3	7.8	97.8	110.7	107.7	113.2	123.1	7.4	0.210	0.4200	0.750
TPY	45.3		37.4	40.5	39.7	43.1	2.7	21.5^a^	1.9^b^	1.9^b^	1.0^b^	1.0^b^	2.5	0.001	< 0.001	0.003
PY	0.93		0.72	0.75	0.72	0.75	1.5	0.92^b^	6.6^a^	7.8^a^	5.3^ab^	6.6^a^	1.4	0.040	< 0.001	0.040
MSPE			0.02^a^	0.01^a^	0.012^a^	0.01^b^	0.001		0.01	0.008	0.008	0.006	0.001	0.004	< 0.001	0.910
RMSPE			0.11^a^	0.10^ab^	0.10^ab^	0.08^b^	0.005		0.10^a^	0.08^ab^	0.08^ab^	0.07^b^	0.004	< 0.001	< 0.001	0.960
AIC			−36.2^a^	−16.8^b^	−11.3^b^	−9.5^b^	1.9		−46.6^a^	−23.3^b^	−14.5^c^	−13.9^c^	1.8	< 0.001	< 0.001	0.210
BIC			−31.1^a^	−14.4^b^	−10.3^b^	−9.3^b^	1.8		−41.5^a^	−20.9^b^	−13.6^b^	−13.6^b^	1.7	< 0.001	< 0.001	0.173
r			0.90**	0.87**	0.86**	0.95**			0.85**	0.68**	0.64**	0.79**				
	Wilmink															
	Typical							Atypical						***P***-value		
	Actual	7D		14D	21D	28D	SEM	Actual	7D	14D	21D	28D	SEM	Interval	Shape	Interval × Shape
TMY	92.9	95.9		100.6	101.5	109.4	7.72	90.3	104.3	99.2	106.1	111.4	6.3	0.07	0.61	0.93
TPY	49.8	37.9		44.62	43.76	48.67	2.67	23.9^a^	1.0^b^	1.8^b^	1.0^b^	1.00^b^	2.2	< 0.01	< 0.01	< 0.01
PY	0.88	0.67		0.69	0.68	0.69	0.06	0.95	1.1	1.2	1.2	1.19	1.2	0.63	< 0.01	< 0.01
MSPE		0.017^a^		0.016^ab^	0.015^ab^	0.012^b^	0.001		0.012	0.011	0.010	0.008	0.001	0.03	< 0.01	0.99
RMSPE		0.12		0.11	0.11	0.09	0.006		0.10^a^	0.09^ab^	0.09^ab^	0.08^b^	0.004	< 0.01	< 0.01	0.97
AIC		−32.5^a^		−15.9^b^	−9.3^bc^	−8.4^c^	2.1		−43.1^a^	−20.0^b^	−13.0^bc^	−11.8^c^	1.7	< 0.01	< 0.01	0.19
BIC		−27.6^a^		−13.5^b^	−8.4^b^	−8.2^b^	2.0		−38.0^a^	−17.7^b^	−12.1^b^	−11.6^b^	1.6	< 0.01	< 0.01	0.19
r		0.90***		0.89***	0.89***	0.88***			0.96***	0.79***	0.95***	0.95***				

*TMY* total milk yield, *PY* peak yield, *TPY* time to peak yield, *MSPE* mean square of prediction error, *RMSPE* root-mean-square prediction error, *AIC* Akaike´s Information Criterion, *BIC* Bayesian´s Information Criterion. Statistical significance for the correlation analysis (r) between actual and predicted total milk yield is given as: * *P* < 0.05; ** *P* < 0.01; *** *P* < 0.001

**Table 3:** Goodness of fit evaluation of two mechanistic models using several milk sampling intervals of F1 dairy sheep

	Dijsktra														
	Typical						Atypical						*P*-value		
	Actual	7D	14D	21D	28D	SEM	Actual	7D	14D	21D	28D	SEM	Interval	Shape	Interval*Shape
TMY	96.9	107.1	106.5	113.2	118.5	13.7	86.6	109.0	112.6	126.6	106.4	11.4	0.19	0.98	0.84
TPY	45.1	45.0	46.1	45.7	47.5	3.7	23.9^a^	7.8^b^	8.9^b^	10.7^a^	22.9^a^	2.5	< 0.001	< 0.001	0.04
PY	0.94	0.79	0.77	0.84	0.81	3.8	0.91	5.2	6.4	12.0	5.54	3.07	0.37	0.01	0.59
MSPE		0.013	0.012	0.009	0.008	0.006		0.012^a^	0.013^a^	0.024^a^	0.074^b^	0.006	< 0.001	< 0.001	0.001
RMSPE		0.11	0.10	0.09	0.08	0.013		0.10^a^	0.10^a^	0.11^a^	0.18^b^	0.011	0.001	0.002	< 0.001
AIC		−37.4^a^	−17.7^b^	−11.2^b^	−14.5^b^	3.1		−43.0^a^	−19.7^b^	−11.1^bc^	−8.8^c^	2.4	< 0.001	0.87	0.203
BIC		−31.1^a^	−14.6^b^	−10.6^b^	−13.9^b^	3.0		−36.8^a^	−16.9^b^	−10.3^b^	−9.7^b^	2.3	< 0.001	0.61	0.28
r		0.99**	0.99**	0.98**	0.96***			0.56***	0.59***	0.22*	0.07				
	Pollott														
	Typical						Atypical						*P*-value		
	Actual	7D	14D	21D	28D	SEM	Actual	7D	14D	21D	28D	SEM	Interval	Shape	Interval*Shape
TMY	96.9	102.8	102.5	100.0	102.7	7.4	86.6^b^	105.8^b^	112.9^b^	114.4^b^	118.7^a^	6.5	0.01	0.17	0.26
TPY	45.1	34.4	37.2	37.1	37.1	4.3	23.3^a^	4.0^b^	13.3^ab^	7.0^b^	13.7^ab^	3.7	0.002	0.001	0.66
PY	0.94^a^	0.70^b^	0.68^b^	0.70^b^	0.69^b^	0.38	0.91^a^	0.66^b^	0.72^b^	0.71^b^	0.76^b^	0.34	0.001	0.72	0.54
MSPE		0.017	0.014	0.014	0.011	0.001		0.019	0.019	0.018	0.019	0.001	0.31	0.004	0.56
RMSPE		0.12^a^	0.11^ab^	0.10^ab^	0.09^b^	0.006		0.13	0.13	0.12	0.13	0.006	0.02	0.001	0.16
AIC		−32.3^a^	−18.1^b^	−16.2^b^	−29.1^ab^	3.8		−33.1^a^	−13.1^b^	−8.4^b^	−4.7^b^	3.3	< 0.001	< 0.001	0.005
BIC		−29.2	−15.5	−15.4	−29.2	3.7		−27.6^a^	−10.2^b^	−6.9^b^	−4.2^b^	3.3	< 0.001	< 0.001	0.005
r		0.90***	0.89***	0.89***	0.88***			0.98***	0.96***	0.97***	0.95***				

*TMY* total milk yield, *PY* peak yield, *TPY* time to peak yield, *MSPE* mean square of prediction error, *RMSPE* root-mean-square prediction error, *AIC* Akaike´s Information Criterion, *BIC* Bayesian´s Information Criterion. Statistical significance for the correlation analysis (r) between actual and predicted total milk yield is given as: * *P* < 0.05; ** *P* < 0.01; *** *P* < 0.001

showed that the Wood model had a better ability to fit typical curves based on r and estimated values of TMY, TPY and PY compared to atypical curves. On the one hand, the Wilmink model fitted atypical curves better in most of the sampling intervals. Both empiric models, however, showed difficulties in estimating TPY and PY in atypical curves. This lack of fit was more evident for the Wood model, which underestimated TPY, overestimated PY and had the lowest values of r (0.64) between the actual and predicted TMY. Nevertheless, the values of MSPE, RMSPE, AIC and BIC to fit atypical curves were significantly lower (*P* = 0.0001) using the Wood and Wilmink models (Table 2).

The goodness of fit of the mechanistic models was also influenced by the shape of the lactation curve (Table 3). The Dijkstra model showed the best ability to fit typical curves with the highest values of r (0.96 to 0.99) and the lowest values of MSPE, RMSPE, AIC and BIC in most of sampling intervals. However, the Dijkstra model had some difficulties in fitting atypical curves, with the lowest values of r among the empirical and mechanistic models.

**Fig. 2:**
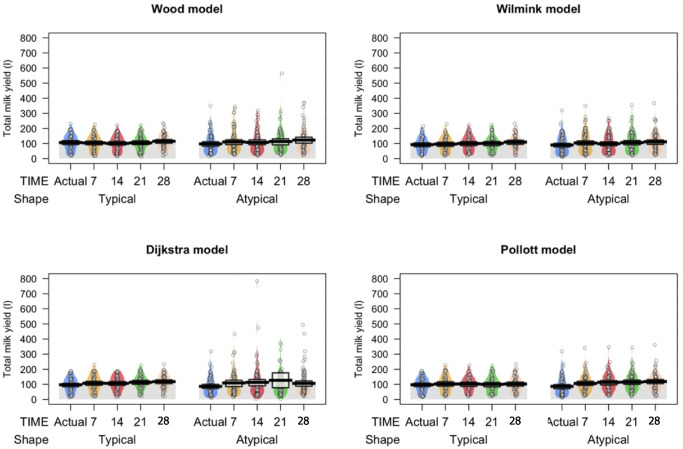
Pirate plots of actual and estimated total milk yield (L) by sampling interval, shape of lactation curve and mathematical model applied to F1 dairy sheep lactations

In addition, this model underestimated TPY, overestimated PY and showed several outliers estimates of TMY to fit atypical curves (Fig. 2). The Pollott model has an adequate performance to fit typical curves according to most goodness of fit criteria. These mechanistic models showed the best fit of atypical curves with the larger values of r (> 0.96) and good estimates of PY, TPY (Table 3) and TMY (Fig. 2).

### Sampling intervals

The results of the current study showed a significant effect of sampling intervals on the ability of empirical and mechanistic models to fit lactation curves of dairy ewes (Tables 2 and 3). However, the level of fitting performance depended on the mathematical model and the estimated lactation characteristic. For TMY, the four models predicted these characteristics with similar accuracy in most sampling intervals (Tables 2 and 3). Only the estimates of TMY by the Dijkstra model in the atypical lactation curve showed significant differences between the 28D and the rest of the sampling intervals, and the actual data. This is also seen in Fig. 2, where the Dijkstra estimated data are outside the smoothed density, which is larger compared to the rest of the models.

There were no significant differences between actual and estimated values of TPY for typical curves in all milk recording systems. On the other hand, the TPY of atypical curves was underestimated by the empirical model in all sampling intervals (Table 2). The data of the 28D sampling intervals fitted by the Dijkstra model showed a good estimation of the TPY of atypical curves. Also, the 14D and 28D sampling intervals fitted by the Pollott model gave estimates of TPY similar to the actual values of atypical curves (Table 3). The sampling intervals had no significant effect on the estimation of TPY in typical curves fitted by the Wood, Wilmink and Dijkstra models. The Pollott model underestimated the PY of typical curves in all sampling intervals. The Wood model estimated the PY of atypical curves better when fitted to the 21D data. Finally, the Pollott model underestimated the PY of atypical curves in all sampling intervals (Table 3).

**Fig. 3:**
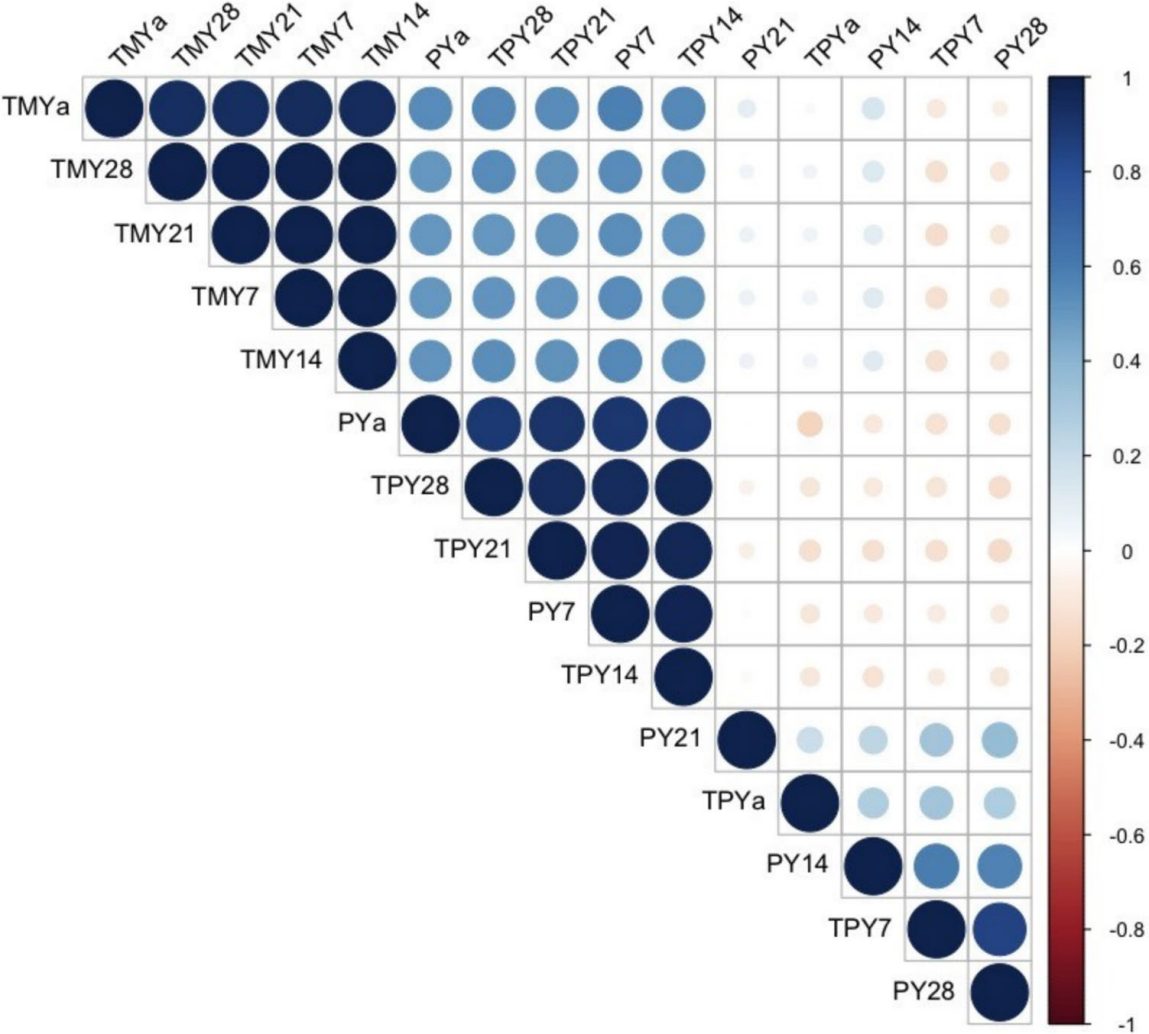
Correlogram between actual and estimated total milk yield (TMY), peak yield (PY) and time to peak yield by the Pollott model according to sampling interval (7, 14, 21, 28 days) of F1 dairy sheep. Actual values of total milk yield actual (TMYa), peak yield (PYa) and time to peak yield (TPY) and estimated from different sampling intervals (7, 14, 21 and 28 days)

The goodness of fit criteria revealed that the Wood model had a better fit to the 28D data of typical curves with lower values of MSPE (0.01), RMSPE (0.08) and highest values of r (0.95). Conversely, the better estimates of TMY were shown by the 7D data with the highest values of r (0.85) compared to the other sampling intervals of atypical curves. For the Wilmink model the results are less clear because the values of r, MSPE and RMSPE were similar among the milk sampling intervals. For both shapes of lactation curves, the best performance was obtained when the 7D sampling interval was used for all milk traits (TMY, PY and TPY; Fig. 3). However, the correlogram showed that the Pollott model performed well for all sampling intervals, regardless of the shape of the lactation (Fig. 3).

## Discussion

### Goodness of fit

Contrasting differences in goodness of fit were observed for the same model or sampling interval, depending on the criteria used. This can be explained by the fact that these criteria evaluate fit performance using different mathematical and statistical approaches and only emphasizes a certain aspect of the error characteristics (Chai and Drazler 2014). For example, AIC and BIC select the best model by searching for the smallest actual-estimated discrepancy as measured by the Kullback–Leibler divergence, rewarding parsimony and penalizing the number of parameters (Bozdogan 1987; Wagenmakers and Farrell 2004). However, AIC and BIC scores can only validly compare models using the same dataset, as the inference is conditional on the data at hand (Burnham and Anderson 2004).

On the other hand, MSPE and RMSPE also measure how close the model predictions are to the actual values (accuracy) (Tedeschi 2006). However, these criteria also provide an estimate of the error in the unit of the actual values and can evaluate different prediction procedures regardless of their mathematical structure and are less sensitive to data structure (Chai and Drazle 2014). For this reason, in the current study, although all goodness of fit criteria were used to evaluate the models and sampling intervals, the values of AIC and BIC were more appropriate to compare the performance of the models. In contrast, MSPE and RMSPE were mainly used to compare between sampling intervals and data sets of typical and atypical curves.

### Lack of convergence

Differences in convergence were observed between the models and the sampling intervals, which could be attributed to the mathematical structure of the functions used and the characteristics of the data. The Pollott model showed the higher number of non-fitting lactations due to lack of convergence, probably related to its complexity, as simpler empirical models can quickly converge to an acceptable solution compared to more complex mechanistic models (López et al. 2015). Another likely reason for the lack of convergence is the poor choice of initial values used in the iterative process (Archontoulis and Miguez 2015). However, in the current study, several values of starting values were tested in the nonlinear fitting process with the aim of avoiding convergence failures.

Regarding the structure of the data used to fit the lactation curve models, Wasike et al. (2011) found convergence failures in cow lactations with fewer observations and missing records, attributing these factors to the over-parameterization of the mathematical functions. This is consistent with the results of the present study, where all models showed a higher lack of convergence as the number of TDR decreased. Also, when convergence was not achieved, it is likely that the selected model was not well suited to describe the actual data (Archontoulis and Miguez 2015). In this sense, most lactation curve models have been developed to fit typical curves. Therefore, failure in the iterative process can be related to the shape of the lactation curve. For example, Wasike et al. (2011) reported more convergence failures in cattle breeds with atypical curves (Guernsey, Jersey and Sahiwal) compared to breeds with typical curves (Holstein).

### Shape of lactation curve

The lactations of dairy sheep are characterized by the presence of numerous atypical curves (20—50%) (Cappio-Borlino et al. 1997) due to genetic potential, environmental factors and nutritional management (Angeles-Hernandez et al. 2018b). A study in Mexico, reported that 52.1% of sheep had atypical lactation curves (Angeles-Hernandez et al. 2014a, b). These authors indicated that this high incidence of atypical curves may be related to the nutritional management based on grazing, which limits the supply of energy and protein during early lactation, thus affecting the pattern of milk production. The current study also analyzed lactations of crossbred sheep, which, according to Cappio-Borlino et al. (1997), increase the frequency of atypical curves.

The accuracy of any given model to predict peak of lactation characteristics was affected by the shape of the lactation curve in all models. In Ayrshire, Holstein and Guernsey cows, the prediction accuracy of two models decreased as the number of atypical lactations increased and this was associated with the curve characteristics (curve shape and slope gradient) (Wasike et al. 2011). Therefore, more reliable and robust results could be obtained by comparing parameter values within groups, i.e. for curves with the same shape (Macciotta et al. 2006). The presence of atypical curves reduced the predictive power of the models. This may be related to peak characteristics.

In our study, the models evaluated assumed that maximum milk yield occurs at the start of lactation on the first day postpartum. However, the Pollott model has less difficulty estimating PY and TPY due to its mechanistic origin, which provides greater flexibility in its parameters. As shown in Fig. 1, the Djikstra and Wood model may overestimate the onset of lactation in the worst case. In the empirical models, there is an underestimation of TPY (22.7 vs. 1.3 days), which can be related to the fact that the Wood and Wilmink models assume that maximum milk secretion occurs at the beginning of lactation between days 1 and 2 postpartum (Table 2).

Estimation of PY was a challenge for all models, but especially for the Wood model. According to the second derivative of the Wood function, the absolute value of the parameter *b* controls the magnitude of the curvature of the lactation pattern (Congleton and Everett 1980); that is, if *b* is positive, the curve is concave, while for negative values of *b* the curve is convex (González-Peña et al. 2012). This demonstrates the relationship between the parameter *b* and the type of curve, sometimes producing very wide curvatures. The Wilmink model compared to the Wood model does not overestimate the PY because this model has substantial independence between the first and second part of the curve: there is a lower correlation between *b* and *c* for both types of shapes (González-Peña et al. 2012). In summary, the challenges encountered in predicting PY and TPY in the presence of atypical curves do not invalidate the models, but indicate the need for caution when applying them in systems with a high proportion of atypical lactations. In addition, other models not investigated in this study may be better suited to capture the characteristics of atypical lactations and provide a more accurate representation.

### Sampling interval

The current study investigated the effect of sampling interval on the goodness of fit of a mathematical model applied to sheep lactation curves. The four sampling intervals provided enough information to accurately estimate TMY to fit empirical and mechanistic models. These results align with the acceptable precision (95%) reported in Sicilo-Sarda sheep to estimate TMY using the standard A4 method (30d) compared to the A2 method (15d) (Othmane and Trabelsi 2007). Furthermore, the results highlight the robustness of the Wood, Wilmink and Dijkstra models in successfully modelling lactations for both atypical and typical curves, even with less input data in the 28-day intervals. However, the performance of the fitting curve could be improved by minimizing the variability due to environmental factors or individual differences between sheep. Conversely, the significant overestimation of TMY observed with the Pollott model at the 28-day sampling interval can be attributed to data insufficiency. The limited number of test-day records (TDRs) restricts the applicability of mechanistic models in dairy sheep, as noted by Dijkstra et al. (2010).

The accurate estimation of PY and TPY is fundamental at farm and research level. Adequate estimates of these traits allow calculation of feed requirements, costs associated with nutritional management, evaluation of genetic potential and prevention of metabolic disorders. Peak yield in dairy ewes has been positively correlated (r = 0.73) with TMY (Pollott and Gootwine 2001), higher genetic potential for milk production (Pollott 2004) and higher heritability estimates (0.1—0.4; Pollott and Gootwine 2001). In addition, high PY can increase the negative energy balance in early lactation and promote metabolic disorders and reduced milk production (Dijkstra et al. 2010). For this reason, the estimation of PY and TPY allows the design of management and nutritional strategies to maximize the performance of dairy ewes. In this sense, our results showed that the sampling interval affects the ability of the models to estimate PY and TPY.

For all sampling intervals, the Wood model underestimated PY, which has been previously reported in studies fitting dairy sheep lactations (Dijkstra et al. 2010; Angeles-Hernandez et al. 2014a, b, 2021). The Wood model also showed difficulties in estimating PY and TPY for atypical lactation curves in all sampling intervals. This lack of fit may be related to insufficient information at the beginning of lactation and around PY (Pollott and Gootwine 2000). In the current study, the average of actual value of TPY ranged between 21.5 and 23.9 for atypical lactation curves; therefore, few TDRs are available to estimate the ascending phase parameter of the Wood model (*b* parameter). Our results indicate a higher ability of mechanistic models to estimate PY and TPY in all sampling intervals and a trend to obtain better estimates of these traits when using 14D and 28D sampling intervals.

In general, based on most of the goodness of fit criteria, the accuracy of mechanistic models decreased as the sampling interval increased. This is consistent with the results reported by Crosse et al. (1988) for dairy cows, in which similar sampling intervals to the present study were evaluated to assess the accuracy in estimating TMY. They found that the 7D and 14D sampling intervals were more accurate, with lower absolute deviations of estimated milk yields from actual yields compared to longer sampling intervals. In the same sense, Dijkstra et al. (2010) indicated that the less satisfactory fit of the mechanistic models may be related to the lower number of TDR and longer sampling intervals used on sheep dairy farms, as the standard method for recording sheep milk implies an interval of 30 ± 3 days between TDR (A4) (ICAR 2018). Nevertheless, our results showed that the Pollott model had the best fitting performance for all sampling intervals for atypical lactation curves of dairy sheep.

The better fit of the Pollott model for atypical curves can be explained by the fact that in the current study the three-parameter version of the Pollott model was used. Other versions with more parameters may have more convergence failures and increase the AIC value due to reduced parsimony. In the reduced version of the Pollott model used in this study, its three estimated parameters are related to the decreasing phase of the curve, resulting in a better estimation because all data points in the atypical curve are assigned to this phase. On the other hand, this model showed difficulties in fitting typical curves due to their low number of pre-peak TDRs, which leads to difficulties in accurate estimation (Pollott and Gootwine 2000).

In conclusion, the results of the current study demonstrate that TMY of F1 dairy sheep can be estimated with high accuracy using the proposed empirical and mechanistic models across all sampling intervals considered. However, the estimation of PY and TPY was influenced by the sampling interval, with better estimates obtained for shorter intervals**.** The fitting of both empirical and mechanistic models was also affected by the shape of the lactation curve, with difficulties in estimating PY and TPY in atypical curves. The Dijkstra model provided the best fit for typical curves, while the Pollott model performed better for atypical curves at all sampling intervals. Therefore, the selection and application of lactation curve models should consider the appropriate sampling interval for dairy sheep farms. Although mechanistic models are more sensitive to a small number of TDRs, they offer a biological interpretation of their parameters, making them valuable for research. On the other hand, empirical models are more flexible in the presence of typical curves, easier to implement, and less affected by the number of TDRs, which is highly useful at the farm level. Furthermore, in production systems where environmental influences on lactation curve shape are minimal, the proposed models can be applied with fewer milk production records, reducing data collection costs while maintaining prediction accuracy. This underlines the relevance of our findings to the global dairy sheep industry, providing valuable insights for optimizing model application under different production conditions.

## Data Availability

The data presented in this study are available on request from the corresponding author.
